# Exo-erythrocytic development of *Leucocytozoon* parasites (Haemosporida, Leucocytozoidae) in song thrushes *Turdus philomelos*

**DOI:** 10.1016/j.ijppaw.2023.08.008

**Published:** 2023-08-30

**Authors:** Carolina Romeiro Fernandes Chagas, Mélanie Duc, Tanja Himmel, Vytautas Eigirdas, Herbert Weissenböck, Gediminas Valkiūnas

**Affiliations:** a P. B. Šivickis Laboratory of Parasitology, Nature Research Centre, Akademijos 2, 08412, Vilnius, Lithuania; b Department for Pathobiology, Institute of Pathology, University of Veterinary Medicine Vienna, Veterinaerplatz 1, 1210, Vienna, Austria; c Ventės Ragas Ornithological Station, Marių 24, 99361, Ventė, Lithuania

**Keywords:** *Leucocytozoon*, Meront, Megalomeront, *Plasmodium*, *Haemoproteus*, Pathogenicity, Asynchronous development, Haemosporida

## Abstract

*Leucocytozoon* parasites (Haemosporida, Leucocytozoidae) are haemosporidians whose diversity, exo-erythrocytic development and potential vectors are the least studied. The knowledge about their exo-erythrocytic development and pathogenicity is fragmentary, resulting in an incomplete comprehension of the impact of these parasites on avian hosts. For a long time, *Leucocytozoon* infections were considered benign to wild birds, even though they were virulent in poultry and responsible for some wild bird population declines. This study aimed to investigate the presence of *Leucocytozoon species* exo-erythrocytic stages in song thrushes *Turdus philomelos* using conventional histological techniques (sections stained by H&E) and chromogenic *in situ* hybridization (CISH). Tissues from ten birds (seven naturally infected and three opportunistic samplings) were examined using both methods. Parasite lineages were identified from blood samples using PCR-based techniques. *Leucocytozoon species* meronts were found in five individuals (in four birds using H&E staining protocol, and in three in CISH-treated histological sections). Meronts were found mainly in the kidneys, but some meronts were also present in the lungs. It was possible to observe different maturation stages of meronts in the same bird individual, indicating an asynchronous development. Cytomeres were readily visible in developing meronts. One megalomeront-like structure was present close to a blood vessel in the heart. It was covered with a prominent capsular-like wall. No inflammatory reaction or necrosis was seen in the tissues surrounding the meronts or the megalomeront-like structure. We could confirm the transmission of three *Leucocytozoon* lineages (lTUPHI14, lSTUR1 and lTUPHI13) in Europe, and add evidence of the transmission of two *Plasmodium l*ineages, including *Plasmodium circumflexum* (pTURDUS1), and *Haemoproteus asymmetricus* (hTUPHI01). We call for further research to better understand *Leucocytozoon* parasite exo-erythrocytic development.

## Highlights

• First report of *Leucocytozoon* exo-erythrocytic stages in song thrushes.
• Meronts of *Leucocytozoon* infecting song thrushes are common in the kidneys.
• A megalomeront-like structure of unusual morphology was found in one bird.
• Chromogenic *in situ* hybridization is essential to confirm parasite identity.
• Confirmed the transmission of three *Leucocytozoon* lineages (lTUPHI14, lSTUR1 and lTUPHI13) in Europe.

## Graphical abstract

**Figure:**
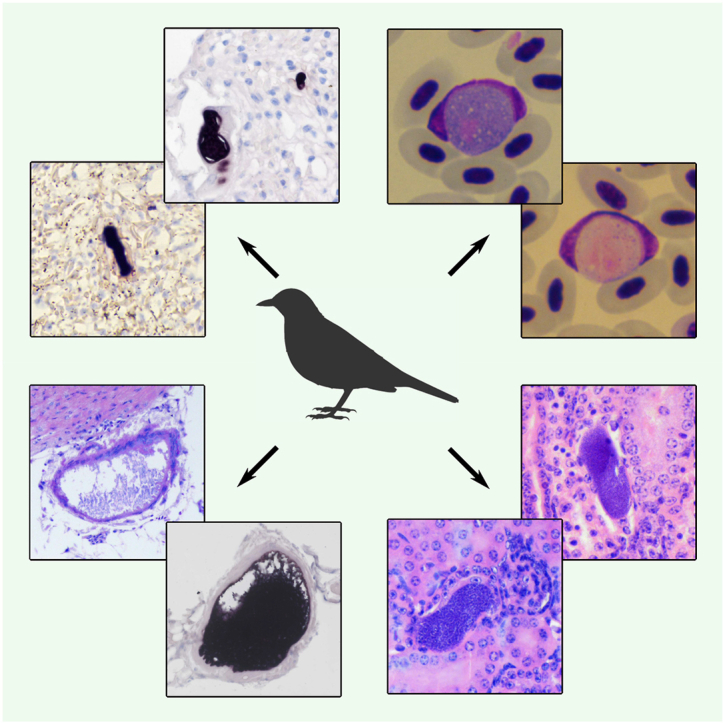

## 1 Introduction

The knowledge about exo-erythrocytic development and pathogenicity of Haemosporida (Apicomplexa) parasites is fragmentary, particularly in wildlife resulting in an incomplete comprehension of their impact on avian hosts (Himmel et al., 2019, 2020, 2021; Valkiūnas and Iezhova, 2017). Avian *Plasmodium* species are better investigated, not only because they were models in human malaria studies for several decades (Braga et al., 2011), but also because it is easier to perform experimental infections using direct transfer of infected blood from a donor bird to recipient avian hosts (Ilgūnas et al., 2019; Palinauskas et al., 2008, 2009). However, *Haemoproteus* and *Leucocytozoon* parasites do not have erythrocytic merogony, making experimental investigations more difficult and requiring vector research (Valkiūnas, 2005). Additionally, meronts of haemosporidian parasites from different genera can be morphologically similar, and difficult to distinguish without the application of modern techniques targeting parasite DNA and/or RNA (Lederer et al., 2002; Peirce et al., 2004; Pennycott et al., 2006; Stidworthy and Greenwood, 2006; Valkiūnas and Iezhova, 2017). These and some other obstacles markedly hampered research on *Leucocytozoon* species (Valkiūnas and Iezhova, 2023). As a result, a few studies addressed the presence of exo-erythrocytic stages of *Haemoproteus* and *Leucocytozoon* in naturally infected wild birds (Valkiūnas and Iezhova, 2017). Recently, with the application of new molecular and histological diagnostic techniques, the parasites of these two genera received more attention (Duc et al., 2020, 2021, 2020; Harl et al., 2022; Hernández-Lara et al., 2021; Himmel et al., 2019, 2019, 2020, 2021, 2019; Ilgūnas et al., 2022). However, *Leucocytozoon* parasites remain one of the haemosporidians on which morphological diversity, exo-erythrocytic development and potential vectors are the least understood and studied (Valkiūnas and Iezhova, 2023).

*Leucocytozoon* infections were identified as being responsible for the death of chickens, turkeys, ostriches, ducks, and geese, and some were confirmed to be involved in the population decline of wild birds (Atkinson and van Riper, 1991; Bennett et al., 1993; Desser, 1967; Khan and Fallis, 1968); even though, for a long time, most haemosporidian parasites, including *Leucocytozoon* species, were considered to be relatively benign to their avian hosts (Bennett et al., 1993). A literature review performed by Forrester and Greiner (2008), indicated that some reports of pathogenicity and mortality of wild birds due to *Leucocytozoon* infections were not well supported by evidence. Recent studies have confirmed that *Leucocytozoon* infections in wild birds can be associated with inflammatory reactions and necroses (Himmel et al., 2020; Ilgūnas et al., 2022), highlighting the necessity for more studies targeting these parasites and to better understand their pathogenicity and pathology in their avian hosts.

To approach these issues, more histological investigations are necessary, which is challenging in wildlife for several reasons. To begin with, the exo-erythrocytic development of given species of *Leucocytozoon* can vary depending on the avian host. A good example is *Leucocytozoon simondi* which was reported to have different development and pathogenicity in Anatidae bird species from different localities (Desser et al., 1978) and from different host species (Desser, 1967; Desser and Ryckman, 1976; Khan and Fallis, 1968). In addition, even among haemosporidian parasites the genus *Leucocytozoon* is the least diverse based on morphological characterizations, with at most 45 morphologically distinguished species (Fecchio et al., 2020; Groff et al., 2022; Valkiūnas and Iezhova, 2023), exo-erythrocytic stages have been investigated, mainly fragmentarily, in only 14 of them. At the same time, the big genetic diversity in this parasite genus, with approximately 1500 cytochrome *b* lineages identified (MalAvi database, http://130.235.244.92/Malavi/accessed in March 2023) (Bensch et al., 2009), also represents a challenge for exo-erythrocytic investigations. This is partly because different parasite lineages might have different patterns of exo-erythrocytic development and pathogenicity (Himmel et al., 2020; Ortiz-Catedral et al., 2019).

*Leucocytozoon* tissue stages in wild birds have been studied in Passeriformes (Himmel et al., 2019, 2020, 2021; Valkiūnas and Iezhova, 2017), with exo-erythrocytic stages being reported in several passerine species, including the blackbird *Turdus merula* (Himmel et al., 2020) and the American robin *Turdus migratorius* (Khan and Fallis, 1970a; Wong and Desser, 1978, 1980, 1981). Even though several song thrushes *Turdus philomelos* individuals have been examined for the presence of *Leucocytozoon* exo-erythrocytic stages, they were never found in this host species. Song thrushes have a large distribution extending all over Europe, the Eastern part of Russia until the Northwest part of Mongolia. Populations are mostly sedentary and some of them can be partial or short-distance migrants. The species breeds in all temperate forests and woodlands, being well adapted to the urban and agricultural landscapes, and presents an increasing population trend being classified as “Least concern” by the IUCN (International Union for Conservation of Nature) Red List of threatened species. Despite that, the song thrush is listed as red in the national United Kingdom Red List due to a decreasing population trend (BirdLife International, 2023). Thus, this study aimed to investigate the presence of *Leucocytozoon* exo-erythrocytic stages in song thrushes using conventional histological techniques (H&E) and chromogenic *in situ* hybridization (CISH).

## 2 Material and methods

### 2.1 Study site, sampling, and microscopic analysis

Song thrushes *T. philomelos* were caught at the Ventės Ragas ornithological station (55°20′38.93″ N, 21°11′34.05” E), Lithuania in September 2020, October 2021 and April 2022. Blood was withdrawn by puncturing the brachial vein and was collected with a heparinized capillary tube. A few drops of blood were used to prepare thin blood smears that were fixed in absolute methanol and stained with a 30% Giemsa solution for 15 min for microscopic analysis, which was performed during fieldwork (Chagas et al., 2020). All blood films were analysed by microscopy following available protocols (Valkiūnas et al., 2008). The remaining blood was stored in SET buffer (0.05 M Tris, 0.15 M NaCl, 0.001 M EDTA, pH 8.0) for further molecular analysis. Seven birds that were positive for *Leucocytozoon* gametocytes were euthanized, and their organs were processed for histological examination (see description below). One individual that was dissected for the investigation of filarioid nematode parasites (the material is being processed and will be published somewhere else) was also included in the study.

Additionally, two other song thrushes found dead in the surrounding areas of the ornithological station in September and October 2021 were collected and kept frozen until dissection (April 2022). They were processed as the other samples and included in the study. During the dissection of these two birds, blood was collected from the heart, a blood film was prepared, and the blood left was stored in SET buffer for further molecular analysis.

Parasitemia was calculated as the percentage of infected cells per 10,000 red blood cells (Godfrey et al., 1987). All microscopic analyses of blood films were performed in fieldwork using an Olympus CX23 light microscope (Olympus, Tokyo, Japan) by experienced parasitologists. Microscopic analyses conducted in the laboratory were performed using an Olympus light microscope BX41 equipped with an Olympus DP12 digital camera and the image software Olympus DP-SOFT (Olympus, Tokyo, Japan). Voucher preparations of gametocytes (accessions 49633NS – 49638NS) were deposited at the Nature Research Centre, Vilnius, Lithuania.

### 2.2 Histological and chromogenic *in situ* hybridization (CISH) examination

During dissection, the brain, heart, lungs, trachea, oesophagus, gizzard, intestine, pancreas, liver, spleen, kidneys, and pectoral muscles were collected and fixed in 10% neutral formalin for 24 h. After that, the organs were washed in distilled water for 1 h and then transferred to 70% ethanol for a period no longer than a month before being processed. At the laboratory, the fixed organs were processed using traditional histological techniques, which consisted in dehydrating the samples in 96% ethanol and isopropanol, followed by a clarification in xylene and embedding in paraffin wax. For each organ, sections of 4 μm were prepared with a microtome, mounted on glass slides, air-dried, stained with hematoxylin-eosin (H&E) and covered with a coverslip (Lillie, 1965). Chromogenic *in situ* hybridization was performed using a specific probe for the detection of *Leucocytozoon* exo-erythrocytic stages (Leuco*18S*) (Himmel et al., 2019). In this case, sections of 1–2 μm were prepared and processed according to the methodology used by Himmel et al. (2019, 2020). Positive controls, which consisted of a sample that was positive for *Leucocytozoon* tissue stages (Himmel et al., 2019), were used on each run.

All microscopic analyses of histological sections were performed in the laboratory using an Olympus light microscope BX41 equipped with an Olympus DP12 digital camera and the image software Olympus DP-SOFT (Olympus, Tokyo, Japan) and an Olympus light microscope BX51 equipped with an Olympus UC90 digital camera and the image software cellSens (Olympus, Tokyo, Japan). Each histological section was screened entirely at low ( × 200) magnification. Voucher preparations of tissue meronts (accessions 49639NS – 49647NS, 49650NS) and megalomeront-like structures (accessions 49648NS and 49649NS) were deposited at the Nature Research Centre, Vilnius, Lithuania.

### 2.3 DNA extraction, PCR and sequencing

The DNA was extracted from blood samples using an ammonium acetate protocol (Sambrook and Russel, 2001). Two nested PCR protocols were used to amplify a fragment of the cytochrome *b* gene (*cytb*) of haemosporidian parasites. The first protocol amplifies ∼480bp of the *cytb* and the first reaction uses the primer set HaemNFI/HaemNR3 and amplifies DNA of *Plasmodium, Haemoproteus* and *Leucocytozoon*. For the nested reaction, the primers HaemF/HaemR2 and HaemFL/HaemR2L were used to amplify the DNA of *Plasmodium/Haemoproteus* and *Leucocytozoon*, respectively (Bensch et al., 2000; Hellgren et al., 2004). The second PCR protocol amplifies ∼545bp of the *cytb* of the genera *Plasmodium, Haemoproteus* and *Leucocytozoon*. The first reaction uses the primer set Plas1F/HaemNR3, and the nested reaction uses the primer set 3760F/HaemJR4 (Pérez-Rodríguez et al., 2013). All primers were used in a 10 pmol/μl concentration. The PCR reactions were performed in 25 μl volumes containing 12.5 μl of Dream taq Master Mix (Thermo Fischer Scientific, Lithuania), 8.5 μl of ultra-pure water, 1 μl of each primer (forward and reverse), and 2 μl of extracted DNA. In the second reaction, 2 μl of the first reaction product was used as a DNA template. The temperature profile of both nested PCR protocols was the same (Bensch et al., 2000; Hellgren et al., 2004; Pérez-Rodríguez et al., 2013). To control for possible contamination and false amplifications, a positive (sample that was positive for each tested parasite), and a negative control (ultra-pure water) were used in every run. Successful amplification was evaluated in a 2% agarose gel, positive samples were purified and sequenced from both directions using corresponding primers with a Big Dye Terminator V3.1 Cycle Sequencing Kit and ABI PRISMTM 3100 capillary sequencing robot (Applied Biosystems, Foster City, CA, USA).

Obtained sequences were evaluated in Geneious Prime (2023).0.1 (https://www.geneious.com, accessed on May 12, 2023), where both sequences were aligned to create a contig sequence. Sequences containing two or more peaks at the same nucleotide position were considered as mixed infections. Parasite lineages were identified using the BLAST tool at the MalAvi database (MalAvi database, http://130.235.244.92/Malavi/accessed in March 2023) (Bensch et al., 2009). All obtained sequences were deposited in GenBank (accession numbers OQ784302, OR472599-OR472613).

## 3 Results

**Fig. 1:**
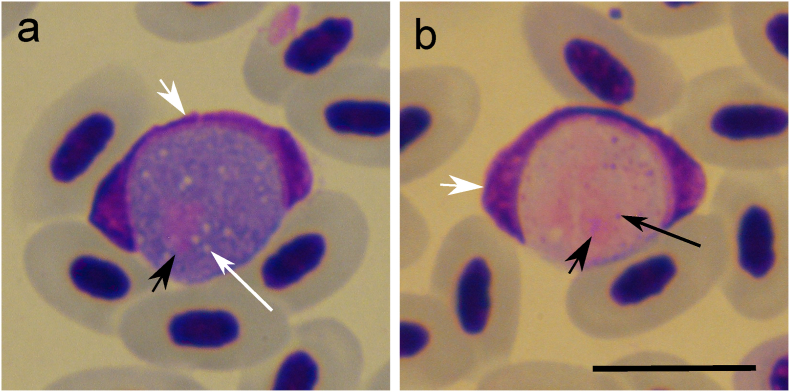
Mature gametocytes of *Leucocytozoon dubreuili* in roundish host cells from the blood of a song thrush *Turdus philomelos* (individual no. 1, cytochrome *b* lineage lTUPHI14). Macrogametocyte (**a**). Microgametocyte (**b**). Short simple white arrows: nuclei of infected host cells. Short simple black arrow: parasite nucleus. Long simple white arrows: vacuoles. Long simple black arrow: volutin granules (small purplish dots). Methanol-fixed and Giemsa-stained. Scale bar 10 μm.

Microscopic analysis of thin blood smears was conducted for nine individuals (nos. 1–8 and 10, see Table 1) because the quality of the thin blood smear collected from individual no. 9 was poor and did not allow such analysis. *Leucocytozoon* parasites were present in seven individuals (nos. 1–7, Table 1), and in all of them, the morphotype present was of *Leucocytozoon dubreuili* with parasitemia varying from 0.005% to 0.09% in different bird individuals. This parasite morphotype is characterized by the deformation of the infected host cell nucleus that assumes a dumbbell shape and with more or less evident thickening at both ends and with the nucleus of the infected cell extending more than half of the circumference of the gametocyte (Fig. 1) (Valkiūnas, 2005). Co-infections with other haemosporidian parasites (*Plasmodium* and *Haemoproteus*) were identified by microscopy in only two individuals (nos. 5 and 6, Table 1). Microfilariae were present in four individuals (nos. 2, 4, 7 and 8, Table 1). Microscopy results and some other data are specified in Table 1.

**Table 1:** Examined song thrushes (*Turdus philomelos*), with the results on blood film microscopy, PCR-based testing, and histology using hematoxylin-eosin (H&E) staining and chromogenic *in situ* hybridization (CISH).

Individual number	Age	Microscopy (*Leucocytozoon* sp. parasitemia)	PCR, cyt*b* lineages and corresponding species	H&E	CISH
1	J	L (0.005%)	pTURDUS1 (*P. circumflexum*) lTUPHI14 (*Leucocytozoon* sp.)	M (kidneys)^a^	BS
2	J	MF, L (0.03%)	pTURDUS1 (*P. circumflexum*) pBT7 (*Plasmodium* sp.) lSTUR1 (*Leucocytozoon* sp.)	M (kidneys)	BS, M (kidneys and lungs)
3	U	L (0.01%)	pTURDUS1 (*P. circumflexum*) lSTUR1 (*Leucocytozoon* sp.)	-^b^	BS, M (kidneys and lungs)
4	J	MF, L (0.01%)	pTURDUS1 (*P. circumflexum*) lSTUR1 (*Leucocytozoon* sp.)	M (kidneys) MM (heart)	BS, M (kidneys), MM (heart)
5	J	P, L (0.06%)	pTURDUS1 (*P. circumflexum*) lTUPHI13 (*Leucocytozoon* sp.) lSTUR1 (*Leucocytozoon* sp.)	M (kidneys)	BS
6	J	H, L (0.01%)	hTUPHI01 (*H. asymmetricus*) lSTUR1 (*Leucocytozoon* sp.)	–	BS
7	J	MF, L (0.09%)	lSTUR1 (*Leucocytozoon* sp.)	–	BS
8	J	MF	–	–	–
9	U	–	pTURDUS1 (*P. circumflexum*)	–	–
10	A	P	Co-infection (*Plasmodium* spp.)	–	–

^a^ Infected organ was indicated in parentheses. ^b^ Negative result. Age: A, adult; J, juvenile; U: unknown. BS: blood stage signal. Parasites found in the blood film: H, *Haemoproteus*; L, *Leucocytozoon*; P, *Plasmodium*. MF, microfilaria of filarioid nematode. Tissue stages: M, meront; MM, megalomeront-like structure.

By PCR-based testing and sequencing, seven birds were positive for *Leucocytozoon* parasites (nos. 1–7, Table 1). Five of them were positive for the *cytb* lineage lSTUR1 (nos. 2, 3, 4, 6, 7), one for lTUPHI14 (no. 1) and one bird had a co-infection of lSTUR1 and lTUPHI13 (no. 5). *Leucocytozoon* parasites were found in co-infections with other haemosporidian parasites in six birds (nos. 1–6). *Plasmodium circumflexum* pTURDUS1 was identified in six birds, and two individuals had a co-infection with different *Plasmodium* lineages (nos. 2, 10). *Haemoproteus asymmetricus* hTUPHI01 was present in only one bird (no. 6). Only two individuals had single infections, one with *Leucocytozoon* lSTUR1 (no. 7) and one with *P. circumflexum* pTURDUS1 (no. 9). One bird was negative for haemosporidian parasites (no. 8). PCR testing results are specified in Table 1.

**Fig. 2:**
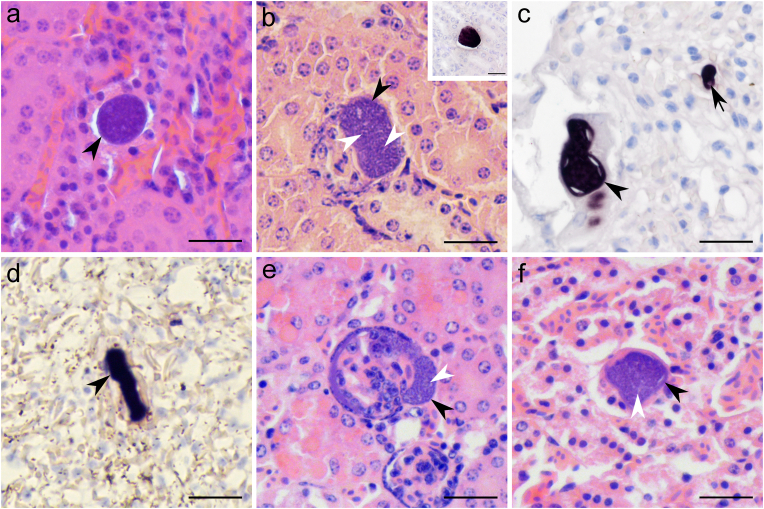
Tissue stages of *Leucocytozoon* parasites in naturally infected song thrushes *Turdus philomelos*, found in hematoxylin-eosin (H&E) stained (**a, b, e, f**) and chromogenic *in situ* hybridization (CISH) treated (insert in **b**, and **c, d**) histological sections. Early meronts in the kidneys of individual no. 1 (cytochrome *b* lineage lTUPHI14) (**a**), and advanced meronts in the kidneys of individual no. 2 (lSTUR1) (**b**) and no. 5 (co-infection of lSTUR1 and lTUPHI13) (**e, f**). Meronts labelled by a *Leucocytozoon*-specific (Leuco*18S*) probe in the kidneys (insert **b**), and lungs of individual no. 2 (lSTUR1) (**c**) and individual no. 3 (lSTUR1) (**d**). Tissue stage developing in the Bowman capsule of a renal corpuscle (**e**). *Leucocytozoon* blood stage labelled by a *Leucocytozoon*-specific probe (**c**). Simple black arrowheads: meronts. Simple white arrowheads: cytomeres (visible as darker stained nuclei surrounded by lighter staining clefts). Short simple black arrow: nucleus of *Leucocytozoon* blood stage. Scale bars 20 μm.

**Fig. 3:**
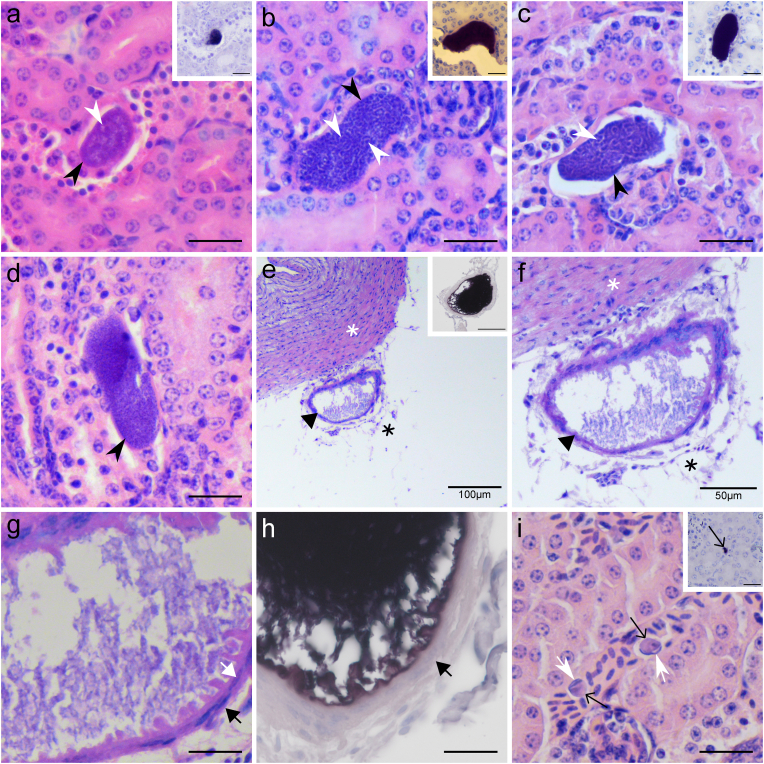
Tissue and blood stages of *Leucocytozoon* parasites in naturally infected song thrushes *Turdus philomelos* (individual no. 4, cytochrome *b* lineage lSTUR1), found in hematoxylin-eosin (H&E) stained (**a-g, i**), chromogenic *in situ* hybridization (CISH) treated (**h** and inserts **a-c, e, i**) histological sections. Early (**a**), advanced (**b, c**) and mature (**d**) meronts in the kidneys. Megalomeront-like structure (**e-h**) located close to a heart blood vessel (white asterisk) within the fat tissue (black asterisk). *Leucocytozoon* gametocytes in the kidneys (**i**). Simple black arrowheads: meronts. Simple white arrowhead: cytomeres (visible as darker stained nuclei surrounded by lighter stained clefts). Black triangle arrowhead: megalomeront-like structure. Short black triangle arrow: capsule-like wall. Short white triangle arrow: host cell nucleus in the capsule-like wall. Short simple white arrows: nuclei of infected host cells. Simple thin black arrows: gametocytes. Scale bars 20 μm, unless indicated otherwise.

In the histological sections stained with H&E, exo-erythrocytic stages were seen in four birds (nos. 1, 2, 4, 5, Table 1). Among these, one was infected with lTUPHI14 (no. 1), two with lSTUR1 (nos. 2 and 4) and one had a co-infection of lSTUR1 and lTUPHI13 (no. 5). In all four H&E-positive birds, tissue meronts were seen only in kidneys (Table 1, Fig. 2a, b, e, f and Fig. 3a–d), they were at different stages of maturation. Early meronts were small structures, with their size ranging from 18 to 24 μm and being roundish to oval, with the presence of condensed chromatin (Fig. 2, Fig. 3a). More developed meronts were also present, this is indicated by the presence of cytomeres, which are formed by the invaginations of the cytoplasm of the infected host cell that breaks down into several parts containing a great number of nuclei (Fig. 2b, e, f, and Fig. 3b and c). These meronts were bigger than the early ones and predominantly elongated, with the biggest length ranging from 18 to 46 μm. Mature meronts were also observed. They were bigger than 50 μm, elongate in form and contained numerous roundish merozoites (Fig. 3d). No capsule-like wall was observed surrounding the renal meronts. No sign of inflammation or necrosis was seen around or close to the meronts.

The number of meronts found in studied birds varied. In individual no. 1, only one early meront was seen (Fig. 2a), while individual no. 4 had several meronts in the kidneys and in different maturation stages (Fig. 3a–d). Also, in individual no. 5 (Fig. 2e and f), different stages of maturation were present. This indicates that the development of these *Leucocytozoon* parasites occurs asynchronously. The host cells infected by *Leucocytozoon* meronts were identified in only one case with the exo-erythrocytic stage located in the epithelial cells of the parietal layer of the Bowman capsule of a renal corpuscle (no. 5, Fig. 2e).

A megalomeront-like structure was seen in one infected bird (no. 4), and it was located close to a large blood vessel and within the fat tissue around the heart (Fig. 3e–h), with 186 μm in the biggest length. The mentioned structure also had a remarkable capsule-like wall, containing numerous nucleated cells of host origin, an atypical appearance for megalomeront walls, which usually do not contain such cells (Fig. 3g–h). The size of this structure suggests that this might be a megalomeront, however, due to the lack of feature that is characteristic of this *Leucocytozoon* developmental stage – the presence of a markedly hypertrophied host cell nucleus or central body of megalomeront - we prefer to refer to it as megalomeront-like structure.

The analysis of CISH-treated histological preparations revealed the presence of blood stages (gametocytes) (Fig. 2, Fig. 3i) in all *Leucocytozoon* PCR-positive birds (nos. 1–7). They were seen within blood vessels and capillaries in almost all examined organs, but at a higher intensity in the kidneys and in the lungs. The presence of meronts in the kidneys was confirmed by CISH in individuals nos. 2–4 (Table 1, Fig. 2, Fig. 3a-c). Additionally, two individuals (nos. 2 and 3) showed meronts in the lungs, but they were not detected in corresponding H&E preparations (Fig. 2c–d). The presence of the megalomeront-like structure was also confirmed by the CISH method (Fig. 3e, h), confirming the presence of *Leucocytozoon* rRNA.

All birds that were PCR negative for *Leucocytozoon* parasites did not present any tissue or blood stages in either H&E or CISH-treated histological preparations (nos. 8–10, Table 1). One of the birds (no. 7) was positive for *Leucocytozoon* by microscopy and PCR-based methods, however, no tissue stages were found, and only blood signals were seen in CISH-treated sections (Table 1).

## 4 Discussion

The main findings of this study are that *Leucocytozoon* meronts of song thrushes develop mainly in the kidneys and asynchronously. Additionally, a megalomeront-like structure was also seen in one of the studied birds. The number of co-infections with other haemosporidian parasites found in the present study is also remarkable (two by microscopy and six by PCR out of 10 birds), but this is not an exception and co-infections are often reported in wild birds (Chagas et al., 2020, 2021; Himmel et al., 2020; Pigeault et al., 2022). The prevalence of *P. circumflexum* pTURDUS1 was high in our study. This parasite species was originally described in the fieldfare *Turdus pilaris* (Kikuth, 1931) and is a generalist parasite that was already reported in over 100 bird species (Malavi database, accessed on 2023-05-25) (Bensch et al., 2009; Valkiūnas, 2005). *Haemoproteus asymmetricus* hTUPHI01 was described in song thrushes, and its prevalence was reported to be close to 15% at the same study site (Valkiūnas et al., 2021).

Six out of nine PCR-positive individuals were juveniles and gametocytes of parasites belonging to three genera (*Leucocytozoon*, *Plasmodium* and *Haemoproteus*) were seen in all these birds (Table 1). The presence of parasite blood stages in juveniles indicates that the recovered parasite lineages complete their life cycle in song thrushes and are transmitted in Europe*.* Previous studies have pointed out the presence of sporozoites infective stages of *H. asymmetricus* hTUPHI01 in salivary gland preparations of different species of *Culicoides* (Bernotienė et al., 2019; Chagas et al., 2022; Žiegytė et al., 2021). The studies targeting vector identification of *P. circumflexum* are still scarce, and its natural vector remains unknown (Herman, 1938; Meyer et al., 1974; Santiago-Alarcon et al., 2012). The vectors of *Leucocytozoon* spp. were determined mainly by experimental research (Santiago-Alarcon et al., 2012). Recently, a few studies showed the presence of *Leucocytozoon* DNA in Simuliidae insects using a PCR-based technique (Jumpato et al., 2019; Pramual et al., 2020; Žiegytė and Bernotienė, 2022), however, no sporozoites were found in the salivary glands preparation (Žiegytė and Bernotienė, 2022) or only a molecular approach was used in the study (Jumpato et al., 2019; Pramual et al., 2020), which does not confirm that *Leucocytozoon* parasite species or lineage can complete its development in the vector. However, due to the difficulties of vector research with black flies (Diptera, Simuliidae) (Valkiūnas and Iezhova, 2023) the number of studies on *Leucocytozoon* vectors are still scarce and should be targeted in future studies.

*Leucocytozoon* lineages found in the present study (lTUPHI14, lSTUR1 and lTUPHI13) have been previously described and are frequently reported in song thrushes, occurring almost exclusively in this host species, except for the lineage lSTUR1 which was also reported in the European robin *Erithacus rubecula* (Passeriformes, Turdidae) (MalAvi database, http://130.235.244.92/Malavi/accessed in March 2023) (Bensch et al., 2009; Santiago-Alarcon et al., 2016). None of these *Leucocytozoon* lineages found in studied birds were linked to morphospecies (MalAvi database, accessed on 2023-05-08) (Bensch et al., 2009), even though in the present study their morphotype resembled *L. dubreuili* (Fig. 1). This parasite was described in Turdidae birds from Vietnam, however, the original description of *L. dubreuili* mentioned only the common name of the type-host, and its species remains unknown (Valkiūnas, 2005). Due to that, this parasite still has no genetic characterization. Three other morphospecies of *Leucocytozoon* were reported to infect Turdidae birds: *Leucocytozoon maccluri*, *Leucocytozoon majoris* and *Leucocytozoon fringillinarum* (Bennett and Cameron, 1975; Lotta et al., 2013). Gametocytes of *Leucocytozoon maccluri* were reported to develop in roundish and fusiform host cells while *L. dubreuili* develops only in roundish host cells. Some questions were raised about the attribution of gametocytes that develop in roundish host cells to *L. maccluri* since such gametocytes were reported in only two birds and the roundish form was seen in only one of them (Valkiūnas, 2005). Thus, the original parasite description (Greiner, 1976) might be based on a co-infection of two different parasites – one developing in fusiform host cells and another in roundish host cells. This issue needs further research, as well as molecular characterization of *L. maccluri* which is still absent. Gametocytes of L. *majoris* and *L. fringillinarum* morphotypes only develop in roundish host cells. They can be distinguished due to the changes caused in the nucleus of the infected cells; i.e., in *L. majoris* infections the nucleus of the infected host cell assumes a band-like shape, with approximately the same width along its entire length; while for *L. fringillinarum* the nucleus of the infected host cell assumes a cap-like form, sometimes band-like in shape and the nucleus usually extends less than half of the circumference of the gametocyte (Valkiūnas, 2005). Even though genetic lineages were reported for *L. majoris* and *L. fringillinarum* (Lotta et al., 2013; Perkins, 2008), these lineages were documented in non-type hosts and far away from type localities (in different zoogeographical regions) than in the parasite original descriptions, so further research on molecular characterization of these two widespread *Leucocytozoon* parasites is needed. Detailed discussion about this matter was provided elsewhere (Chagas et al., 2023; Valkiūnas and Iezhova, 2023).

The morphological identification of *Leucocytozoon* parasites is challenging, even for experienced parasitologists. This is mainly due to the presence of a few morphological features that can be used for morphological identification, resulting in parasites with similar morphology, but which might be genetically different (Galen et al., 2018; Harl et al., 2020; Valkiūnas and Iezhova, 2023). Currently, the combination of morphology and molecular analysis is the gold standard methodology for Haemosporida species description (Chagas et al., 2020; Valkiūnas and Iezhova, 2017, 2018, 2023). *Leucocytozoon* species identification could benefit from the analysis of more characteristics of their developmental stages, such as the host cell type inhabited by *Leucocytozoon* gametocytes, the morphological details and location of meronts and megalomeronts in tissues, peculiarities of sporogonic development and vector species, and patterns of development in the vector and avian host tissues (Chagas et al., 2023; Valkiūnas and Iezhova, 2023). However, many of these features are unknown in most of the described species, and the tools for their research need to be better developed and standardized for taxonomical purposes, being essential to encourage future studies involving these aspects of *Leucocytozoon* species biology.

The exo-erythrocytic part of the life cycle of *L. dubreuili* was experimentally investigated using sporozoite-induced infection in the American robin (Khan and Fallis, 1970a; Wong and Desser, 1978, 1981). After the sporozoites were inoculated in birds, they can follow three different paths of development. In the first one, they will infect parenchymal cells of the liver forming the first generation of hepatic meronts. Part of the merozoites released from these meronts, will infect blood cells (parasites can be seen in thin blood films between 3 and 4 days post-infection), and another part will initiate secondary merogony in the liver and kidneys. In the second path, the sporozoites will infect cells of the renal proximal tubules and initiate the first generation of meronts in the kidneys. Meronts induced by sporozoites and by the first-generation of hepatic meronts appear at the same time and their merozoites will infect blood cells and develop into gametocytes (parasites can be seen in the blood between 7 and 9 days post-infection). In the third path, part of the inoculated sporozoites infect parenchymal cells of the liver and develop into oocyst-like meronts, that will form elongated merozoites, being probably responsible for the relapse of infections during spring. In our study, meronts were found only in the kidneys (H&E and CISH-treated histological sections) and in the lungs (CISH-treated histological sections) of infected birds, but no tissue stage was found in the liver or any other organs. According to Khan and Fallis (1970a) and Wong and Desser (1981, 1978), tissue stages were seen in the kidneys already 90 h post-infection and they were visible longer than a month, but liver meronts were not so common anymore seven days post-sporozoite-induced infection. Additionally, lung meronts were also seen eleven days post-infection (Khan and Fallis, 1970a). Thus, some similarities in exo-erythrocytic development and morphology of tissue stages described in previous experimental studies were reported in the examined birds during our study. Mainly, the presence and the similar size of renal meronts, the absence of a capsule-like wall around renal meronts and the elongate or oval shape of the renal meronts (Khan and Fallis, 1970a). The fact that no meronts were found in the liver but they were seen in the kidneys and the lungs in our study, and that all bird individuals with exo-erythrocytic stages were juveniles, reinforces the hypothesis that these birds were infected in the hatching year with *Leucocytozoon* parasites. It is worth mentioning that the development of *Leucocytozoon* species meronts in thrushes follows the stage of cytomeres formation, in which merozoites develop (Fig. 2b, e, f and 3b-d). That is in accordance with published data (Khan and Fallis, 1970a, 1970b; Wong and Desser, 1978, 1981). It seems that cytometere formation is a general pattern during meront maturation in *Leucocytozoon* parasites (Valkiūnas, 2005; Valkiūnas and Iezhova, 2023).

We observed an asynchronous development of *Leucocytozoon* parasites in one of the studied birds, and this characteristic was reported before (Khan and Fallis, 1970a; Wong and Desser, 1978, 1981). Meronts in different stages of development were seen in the kidneys of individual no. 4 (Fig. 3a–d). This individual had a bigger number of meronts than the other studied birds. Noteworthy, the megalomeront-like structure was detected in the same individual (Fig. 3e–h, Table 1). However, one of the main morphological features that characterize the megalomeronts - the presence of a markedly hypertrophied host cell nucleus (Valkiūnas, 2005) - was not observed. Furthermore, the inside structure of the megalomeront-like parasite could not be recognized based on available data. These features raised questions concerning the identity of this structure and if it is indeed a megalomeront. With the CISH-treated section, we could confirm the parasite identity on the genus level, but the lineage and developmental stage of this structure is unclear. It might be still an unknown stage of *Leucocytozoon* parasite development, so worth further investigation. Some recent studies demonstrated megalomeront-like structures of the *Leucocytozoon* parasite (lineage lSTAL5) in owls (Ilgūnas et al., 2022). In that study, numerous *Leucocytozoon* sp. meronts aggregated in the lumen of blood vessels, resulting in a big megalomeront-like structure. With the application of CISH and immunohistochemistry methodologies, the authors could show that this structure was not a megalomeront but consisted of multiple meronts which were developing in endothelial cells of a blood vessel (Ilgūnas et al., 2022). These findings show the limited knowledge of exo-erythrocytic development of leucocytozoids and reinforce the importance of combining different diagnosis techniques to investigate exo-erythrocytic development of haemosporidian parasites, which is especially true in cases of co-infections with other haemosporidian parasites, that are commonly found in nature (Clark et al., 2016; Marzal et al., 2008; Reis et al., 2021; Rooyen et al., 2013; Soares et al., 2016).

The presence of megalomeronts was not reported during *L. dubreuili* exo-erythrocytic developmental studies (Khan and Fallis, 1970a, 1970b; Wong and Desser, 1978, 1981), and some authors mentioned that *L. dubreuili* might not develop megalomeronts (Atkinson and van Riper, 1991). The fact that only one megalomeront-like structure was seen in the present study, does not rule out the possibility of megalomeronts developing in song thrushes. Additionally, the pathogenicity of *Leucocytozoon* parasite species is associated with the presence of megalomeronts (Atkinson and van Riper, 1991). Even though we did not detect necrosis or inflammatory reactions around the meronts and megalomeront-like structure in our samples, that does not mean that these parasites cannot be harmful to their hosts. However, as these *Leucocytozoon* lineages are more or less host-specific (Harl et al., 2020), they might not be highly pathogenic for adapted song thrushes. Further studies are needed on these issues.

The identification of tissue meronts of parasite species belonging to the order Haemosporida (and other parasites of phylum Apicomplexa) is challenging, mainly because certain stages of development can be very similar even for different parasite genera, and tissue stages of unusual morphology and location might occur (Ilgūnas et al., 2022; Lederer et al., 2002; Peirce et al., 2004; Pennycott et al., 2006; Stidworthy and Greenwood, 2006; Valkiūnas and Iezhova, 2017). Furthermore, recent studies have shown that the morphological features of exo-erythrocytic development at the genus (and in some cases at species) level are different in haemosporidian parasites (Himmel et al., 2019). *Haemoproteus asymmetricus* (hTUPHI01) exo-erythrocytic stages are characterized by the formation of clusters of several megalomeronts of different sizes (Himmel et al., 2019). *Plasmodium circumflexum* exo-erythrocytic stages findings include the presence of meronts in the capillaries of the brain, liver, and kidneys (Huchzermeyer and Van Der Vyver, 1991). The application of CISH methodology using specific probes which target parasites of different genera, species or even lineages thus is essential and should be encouraged in future studies on the exo-erythrocytic development of haemosporidian parasites.

## 5 Conclusion

Meronts of *Leucocytozoon* parasites infecting song thrushes are common in kidneys, and they show asynchronous development. A megalomeront-like structure of unusual morphology was found in one bird, and certainly belongs to *Leucocytozoon* parasites, indicating the possibility of an undescribed path of exo-erythrocytic development for these parasites. Because of a single record and the lack of the main megalomeront characteristic features it was not possible to confirm if this is indeed a megalomeront, or if it represents a different stage of development of avian *Leucocytozoon* parasites. We could confirm the transmission of three *Leucocytozoon* (lTUPHI14, lSTUR1 and lTUPHI13), two *Plasmodium* (pTURDUS1 and pBT7) and one *Haemoproteus* (hTUPHI01) lineages in Europe. More studies on tissue stages of *Leucocytozoon* parasites should be done in the future to better understand parasite exo-erythrocytic development.

## Funding source declaration

This study was funded by the European Social Fund and the Research Council of Lithuania [grant number 09.3.3-LMT-K-712-23-0010]. Some of this research was also funded in whole, or in part, by the Austrian Science Fund (10.13039/501100002428FWF) [grant P 33480]. For the purpose of open access, the author has applied a CC BY public copyright license to any Author Accepted Manuscript version arising from this submission.

## Ethics statements

This study complies with the current laws of Lithuania and was approved by the Lithuania Environmental Protection Agency, Vilnius (2020 04 08 Nr. (26)-A4E-2892; 2021 05 05 N. (26)-SR-96, and 2022 04 28 Nr. (26)-SR-152).

## Declaration of competing interest

In regard to the manuscript “*Exo-erythrocytic development of Leucocytozoon parasites (Haemosporida, Leucocytozoidae) in song thrushes Turdus philomelos*”, submitted to the International Journal for Parasitology: Parasites and Wildlife, all authors declare that they have no conflicts of interest.

This statement is included in the manuscript file that was reviewed and approved by all the authors.

On behalf of all the authors.
